# Characterizing biomarkers of ageing in Singaporeans: the ABIOS observational study protocol

**DOI:** 10.1007/s11357-025-01511-1

**Published:** 2025-01-17

**Authors:** Jessica K. Lu, Weilan Wang, Janjira Soh, Elena Sandalova, Zhi Meng Lim, Santosh Kumar Seetharaman, Jing-Dong Jackie Han, Desmond B. Teo, Brian K. Kennedy, Jorming Goh, Andrea B. Maier

**Affiliations:** 1 Centre for Healthy Longevity, National University Health System, Singapore, Singapore; 2 Healthy Longevity Translational Research Program, Yong Loo Lin School of Medicine, National University of Singapore, Singapore, Singapore; 3 Department of Medicine, Yong Loo Lin School of Medicine, National University of Singapore, Singapore, Singapore; 4 Healthy Ageing Programme, Alexandra Hospital, Singapore, Singapore; 5 Division of Geriatric Medicine, National University Hospital, Singapore, Singapore; 6 Peking-Tsinghua Center for Life Sciences, Academy for Advanced Interdisciplinary Studies, Center for Quantitative Biology (CQB), Peking University, Beijing, China; 7 Peking University Chengdu Academy for Advanced Interdisciplinary Biotechnologies, Chengdu, China; 8 Fast and Chronic Programmes, Alexandra Hospital, Singapore, Singapore; 9 Division of Advanced Internal Medicine, National University Hospital, Singapore, Singapore; 10 Department of Biochemistry, Yong Loo Lin School of Medicine, National University of Singapore, Singapore, Singapore; 11 Department of Physiology, Yong Loo Lin School of Medicine, National University of Singapore, Singapore, Singapore; 12 Department of Human Movement Sciences, @AgeAmsterdam, Faculty of Behavioural and Movement Sciences, Amsterdam Movement Sciences, Vrije Universiteit, Van Der Boechorstsraat 7, 1081 BT Amsterdam, The Netherlands; 13 NUS Academy for Healthy Longevity, Yong Loo Lin School of Medicine National University of Singapore, Singapore, Singapore

**Keywords:** Aging, Biological clocks, DNA methylation, Geroscience, Biomarkers

## Abstract

Ageing is the primary driver of age-associated chronic diseases and conditions. Asian populations have traditionally been underrepresented in studies understanding age-related diseases. Thus, the Ageing BIOmarker Study in Singaporeans (ABIOS) aims to characterise biomarkers of ageing in Singaporeans, exploring associations between molecular, physiological, and digital biomarkers of ageing. This is a single-centre, cross-sectional study that recruits healthy community-dwelling adults (≥ 21 years) from three different ethnic groups (Chinese, Malay, and Indian). Molecular biomarkers of ageing include multi-omics approaches, such as DNA methylation analysis and metabolic and inflammatory proteomic profiling in blood, saliva, and stool. Physiological biomarkers of ageing include bone density, body composition, skin autofluorescence, arterial stiffness, physical performance (e.g., muscle strength and flexibility), cognition, and nutritional status. Digital biomarkers of ageing include three-dimensional facial morphology and objectively measured physical activity. Additional measures, such as habitual physical activity, dietary patterns, and medical history, are also examined. The associations between the molecular, physiological, and digital phenotypes will be explored. This study is expected to generate a comprehensive profile of molecular, physiological, and digital biomarkers of ageing in Chinese, Malay, and Indian populations in Singapore. By integrating diverse age-related biomarkers, clinical indicators, and lifestyle factors, ABIOS will offer unique insights into the ageing process specific to Southeast Asian populations. These findings can help identify markers of biological ageing, uncover ethnic-specific patterns, and reveal modifiable lifestyle factors for healthier ageing. The results could inform evidence-based health policies, personalized interventions, and future cross-ethnic comparative studies to enhance understanding of ageing biology across diverse populations.

**Supplementary Information:**

The online version contains supplementary material available at 10.1007/s11357-025-01511-1.

## Introduction

By 2050, one in every six individuals worldwide will be over 65 years old, leading to an increasing number of older adults globally relative to adults in the workforce [1]. Asia’s population is ageing faster than that of any other world region [2]. In Singapore, where the rate of ageing is particularly accelerated, 24% of the population is expected to be older than 65 years by 2030 [3]. Although governmental initiatives have been implemented to develop care solutions for older individuals [4] and to enable residents in Singapore to practise healthier lifestyle habits (e.g., the National Steps Challenge [5] and Nutri-Grade labelling [6]), the healthspan-lifespan gap is around 10 years, indicating that individuals have to endure a significant period of poor health that also stresses healthcare systems [7]. Thus, physiological and physical characterisation of the adult population is important to inform healthcare policies to guide early interventions.

Biological ageing is the primary driver of age-associated chronic diseases [8]. Higher biological age relative to one’s chronological age is characterised by reduced or impaired function of multiple organs required for the function of many physiological systems, such as the cardiovascular, metabolic, and musculoskeletal systems [9]. Differences in the rate of biological ageing can already be detectable at midlife [10]. Measuring biomarkers of ageing (molecular, physiological, and digital) will enhance understanding of the biological processes underlying ageing and their consequences [9]. Age-related changes can be observed at the (i) molecular and cellular levels, such as through DNA methylation patterns (molecular), with (ii) clinical assessments, such as for cardiovascular and physical functions (physiological), and by using (iii) digital devices, such as measuring physical activity and facial age (digital) [9].

Most knowledge about age-related changes has come from clinical studies conducted in Caucasian populations [11, 12]. However, genetic and lifestyle differences between Asian and Caucasian populations are associated with variations in biological age and health risk factors [13–16]. These differences have prompted further investigation into the physiological processes underlying ageing and its consequences in Asian populations [17–19]. Despite this need, a significant gap remains in the availability of relevant datasets for Asian populations. Singapore’s unique multi-ethnic population includes individuals of Chinese, Malay, and Indian ethnicities, and thus provides a valuable study resource to better understand the phenotypes associated with ageing in Asian populations [20].

The Ageing BIOmarker Study in Singaporeans (ABIOS) aims to describe molecular, physiological, and digital biomarkers of ageing in Singaporeans. The secondary aim is to explore factors (e.g., ethnicity, lifestyle, etc.) that are associated with these biomarkers of ageing.

## Methods

### Study design and setting

The Ageing BIOmarker Study in Singaporeans (ABIOS) is a single-centre observational and cross-sectional study of community-dwelling Chinese, Malay, and Indian adults conducted at Alexandra Hospital (AH, Singapore). Participants were recruited from the general Singapore population through diverse outreach efforts to ensure representation across ethnic minorities and age groups. These efforts include study advertisements distributed to the public, targeted outreach to various employers and occupational groups, and language-specific recruitment drives conducted in places of worship, religious organizations, and community associations throughout Singapore.

This study was approved by the National Healthcare Group Domain-Specific Review Board (NHG DSRB Ref: 2019–00388) with all ethical guidelines adhered to under the Declaration of Helsinki [21]. This study has been registered in ClinicalTrials.gov with the identifier NCT06555978. Written informed consent is conducted with all participants before the first study visit commences.

### Participant criteria and sample size

Eligible participants (up to *N* = 420) are community-dwelling adults aged 21 years and older, of Chinese, Malay, or Indian ethnicity, who have lived in Singapore for at least five consecutive years, and recruited between March 12, 2021, and July 1, 2024. Participants must be apparently healthy, non-smokers, and have no more than one health condition if ≥ 45 but < 65 years and can have more than one health conditions if ≥ 65 years. Participants must have all health conditions controlled by medication, if present, to be eligible. Detailed inclusion and exclusion criteria are outlined in Table 1.

**Table 1:** Inclusion and exclusion criteria

Inclusion criteria	Exclusion criteria
≥ 21 years of age	Body mass index (BMI) ≥ 30 kg/m^2^
Chinese, Malay, or Indian and has lived in Singapore for at least 5 consecutive years	Pre-existing or history of major cardiovascular disease (e.g., coronary artery disease, heart failure, stroke, peripheral vascular disease)
Apparently healthy and non-smokers	Any metal implants (excluding dental implants) in the body
If ≥ 45 but < 65 years, having only 1 condition and it must be medication-controlled (if any): • Hypertension • Hyperlipidemia • Hyperglycemia • Osteopenia/osteoporosis • Osteoarthritis • Type 2 diabetes	Pre-existing or history of cancer or chronic obstructive pulmonary disease (COPD)
If ≥ 65 years, having any condition and they must be medication-controlled (if any): • Hypertension • Hyperlipidemia • Hyperglycemia • Osteopenia/osteoporosis • Osteoarthritis • Type 2 diabetes	Females currently pregnant or planning pregnancy in the next 6–12 months

### Screening

After indication of interest, individuals are screened over a phone call for eligibility (Table 1) to participate based on previous and current medical history. Individuals are excluded if they are unable or unwilling to provide written informed consent, without a legal proxy to consent, or are ineligible due to any of the exclusion criteria. If they are eligible according to the screening inclusion criteria, they will be invited for the study visits.

### Study visits

**Fig. 1:**
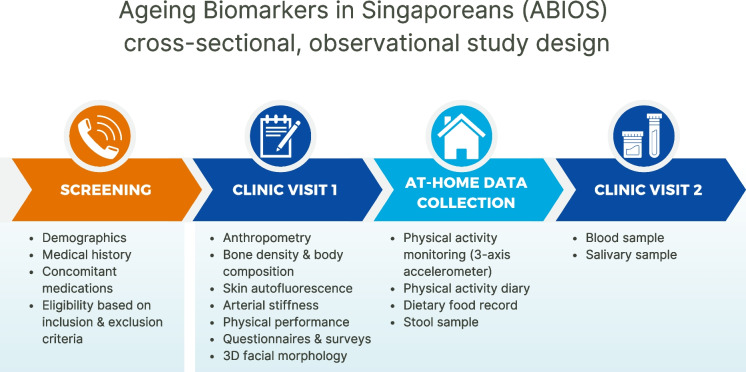
Workflow of ABIOS study recruitment process and study design. Screening of interested individuals is conducted via phone call (*Screening*). Eligible participants complete informed consent before commencing the indicated assessments in the clinic during the first visit (*Clinic Visit 1*). They will then bring home an activity monitor, a 7-day physical activity diary, a 3-day dietary food record, and a stool collection kit for remote data collection (*At-Home Data Collection*). They will return to the clinic during the second visit for collection of blood and saliva and return of the activity monitor, activity diary, dietary food record, and the stool sample collected the morning of the visit (*Clinic Visit 2*). The first and second visits are conducted approximately 11 to 21 days apart

Eligible participants attend the two study visits at AH. Written informed consent is obtained from all participants prior to data collection. A pre-trial questionnaire is administered to check if the participants feel fit to perform the study assessments at the beginning of each visit. Resting blood pressure measurement on the right arm using a digital automated sphygmomanometer (Automatic Blood Pressure Monitor HEM-7121, Omron Healthcare) is taken to ensure it is ≤ 160/90 mmHg before commencing assessments. The two visits are conducted approximately 11 to 21 days apart (Fig. 1). During the first visit, anthropometry, bone density and body composition with dual energy X-ray absorptiometry (DEXA), assessments for skin autofluorescence, arterial stiffness, physical performance, and facial morphology image capture are conducted in addition to the health and lifestyle survey, physical activity questionnaire, cognitive performance test, and nutritional assessment (Table 2). Between the first and second visits, the participant is provided with a physical activity monitor, physical activity diary, and a dietary food record for remote data collection. The stool collection kit, along with instructions, is also provided to the participant. They return the physical activity monitor, physical activity diary, food record, and stool sample during their second visit. During the second visit, collection of blood and saliva is conducted (Table 2).

**Table 2:** Overview of assessments and visits

Methodology	1st visit	Home*	2nd visit
Signed Informed Consent	**√**		
*Clinical assessments*			
Anthropometry	**√**		
Body Density and Body Composition (DEXA scan)	**√**		
Skin Autofluorescence (AGE reader)	**√**		
Arterial Stiffness (carotid-femoral pulse wave velocity)	**√**		
Physical Performance	**√**		
3D Facial Ageing image	√		
*Questionnaires & surveys*			
Health & Lifestyle Survey	√		
Modified Pittsburgh Sleep Quality Index questionnaire	√		
SATED questionnaire	√		
Global Physical Activity Questionnaire (GPAQ)	√		
Montréal Cognitive Assessment (MoCA)	√		
FRAIL	***√***		
Mini Nutritional Assessment (MNA)	√		
7-day Physical activity diary		√	
3-day Food record		√	
*Blood parameters*			
DNA methylation			√
Complete blood count			√
Fasting haematological panel			√
Fasting lipid profile			√
Fasting glucose			√
Fasting liver profile			√
Insulin & HbA1c			√
Immune and inflammatory markers			√
*Other samples and assessments*			
Saliva samples			√
Stool samples		√	
Activity monitor		√	

^*^Home indicates remote data collection at home

Abbreviations: *3D*, three-dimensional; *AGE*, advanced glycation end products; *DEXA*, dual-energy x-ray absorptiometry; *HbA1c*, haemoglobin A1c

Physical performance assessments include handgrip strength, 4-m walk, arm curl, chair sit to-stand, back scratch, and eight repetition-maximal leg extension assessments

To minimize dropout rates following the initial visit, several retention strategies were implemented. First, participants receive regular reminders (via WhatsApp or email) before each scheduled visit. Second, flexible scheduling options are offered to accommodate participants’ availability. Additionally, participants are provided with reimbursement vouchers to reduce logistical barriers to and increase incentives for attendance.

### Molecular biomarkers of ageing

#### DNA methylation

DNA will be extracted from de-identified peripheral blood mononuclear cell (PBMC) samples using QIAamp® DNA Mini Kits (QIAGEN Singapore Pte. Ltd.). The quality of the genomic DNA will be assessed using the Qubit™ fluorometer, through A260/A280 and A260/A230 spectrophotometry ratios and the concentrations of DNA isolated. Approximately 1 µg of genomic DNA from each participant will be bisulphite-treated using the Zymo EZ DNA Methylation Kit (Zymo Research Corporation) to convert non-methylated cytosine nucleotides to uracil for subsequent methylation profiling. The Illumina Infinium MethylationEPIC v2.0 BeadChip Kit (the “935 K”) (Illumina, Inc., USA) will be used for high-throughput genome-wide DNA methylation measurement. The 935 K enables reliable and reproducible evaluation of over 935,000 CpG methylation sites, offering extended genomic coverage of gene regulatory regions compared to its 850 K predecessor [22]. The biological age of participants will be analysed using blood-based DNA methylation clocks: Horvath [23], Hannum [24], PhenoAge [25], GrimAge [26], DunedinPoAM [27], and DunedinPACE [12]. The biological age of the participants will be analysed using each clock separately and using the mean biological age derived from the Horvath, Hannum, PhenoAge, and GrimAge clocks.

#### Blood, saliva, and stool parameters

Peripheral blood (up to 52 mL) will be drawn into Vacutainer plain tubes, EDTA tubes, or sodium fluoride tubes from the antecubital vein by a certified phlebotomist, with the participant having fasted overnight and in a seated position. Blood samples are analysed immediately according to well-established protocols for haematology, coagulation, and biochemistry test parameters: complete blood count, haematology panel, lipid panel, glucose, glycated haemoglobin (HbA1c), insulin, and liver protein panel (total bilirubin, aspartate transaminase, alanine transaminase, gamma-glutamyl transferase, total protein, albumin, globulin) (Supplementary Material, Table 1). PBMCs are isolated from whole blood immediately after blood collection using the Lymphoprep™ density gradient medium in SepMate™ PBMC isolation tubes (STEMCELL Technologies Inc., Canada) and cryopreserved in liquid nitrogen until future DNA methylation profiling and immunophenotypic analysis (Supplementary Material, Table 1). The remaining blood samples in the form of frozen buffy coats, plasma, and serum are stored at –80℃ for future analysis, including circulating immune and inflammatory markers (several myokines, cytokines, damage-associated proteins, metabolites as well as several inflammatory biomarkers) (Olink®, Uppsala, Sweden) (Supplementary Material, Table 1). Samples are placed on ice or in a 4℃ fridge until they can be processed, and the maximum time allowed from sample collection to processing is two hours.

Fasted saliva samples are collected from the participants, who are instructed to passively drool into a plastic funnel that is attached to the top of the saliva collection tube, which is pre-filled with guanidine-free, DNA stabilization buffer (Muhdo Health Ltd., Ipswich, UK). Samples are immediately placed on ice until they can be stored in –80℃ freezers. The samples are cryopreserved at –80℃ until future extraction of genomic DNA with the QIAamp® DNA Mini Kit (QIAGEN Singapore Pte. Ltd.) for subsequent metagenomics sequencing to analyse the salivary microbiome as oral microbial signatures were found to associate with age and frailty [28] (Supplementary Material, Table 1).

Stool samples for gut microbiota analyses are collected for metagenomics sequencing to estimate microbial richness and diversity. Participants are provided with an empty stool collection container during the first visit. The stool samples are collected within 24 h of the second visit (e.g., collected the night before and kept in the refrigerator or collected the morning of the second visit and not kept in the refrigerator) and returned in the stool container during the second visit to be stored at –80℃ for future analysis (Supplementary Material, Table 1).

### Physiological biomarkers of ageing

#### Anthropometry

A stadiometer (SECA217, SECA Gmbh&Co KG, Germany) is used to assess height (measured to the nearest 0.1 cm). Weight is assessed using a floor weighing scale (measured to the nearest 0.1 kg) (SECA813, SECA Gmbh&Co KG, Germany). Body mass index (BMI) is calculated using body mass (kg) divided by height (m) squared expressed in kg/m^2^. Waist and hip circumferences are measured twice using a standard measuring tape (SECA201, SECA Gmbh&Co KG, Germany) at the level of the umbilicus (waist) [29, 30] and at the level of the symphysis pubis and the greatest protrusion of the gluteal muscle (hips).

#### Bone density and body composition

Hip and lumbar bone mineral density as well as lean and fat mass are quantified using the fast array mode with dual energy X-ray absorptiometry (DEXA) (Horizon® DXA system, Hologic, Inc., USA). Body composition is expressed as body fat mass and lean mass in absolute (kg) and relative (%) terms (Supplementary Material, Table 2).

#### Skin autofluorescence

Tissue advanced glycation end-products (AGEs) are estimated using the AGE Reader (Diagnoptics Technologies B.V, the Netherlands), which has been validated against skin biopsies and measures skin autofluorescence [31]. Autofluorescence in human skin tissue is measured using 440-nm ultra-violet (UV) light rays (arbitrary units), the wavelength at which AGEs have high absorbance. The measurement is repeated three times, and the average is used.

#### Blood pressure and arterial stiffness

Pulse wave analysis (PWA) and carotid-femoral pulse wave velocity PWV (cfPWV) are measured using a non-invasive pressure waveform diagnostic tool (SphygmoCor-XCEL, AtCor Medical, Australia) with the participant in the supine position [32]. For PWA, after a 15-min rest, brachial and central aortic blood pressures and heart rate are measured using a brachial pressure cuff. For cfPWV, the three-point-subtraction-method was used [32]. The linear distances (in mm) from the carotid artery to the suprasternal notch and from the suprasternal notch to the femoral artery are measured with a standard measuring tape (SECA201, USA) [32]. The carotid and femoral pulses are simultaneously assessed using a tonometer and a thigh pressure cuff, respectively, to record the arterial pressure waveforms. The cfPWV reading is calculated by subtracting the distance between the carotid artery to suprasternal notch from the distance between the suprasternal notch to femoral artery then divided by the pulse transit time (in metres/second) [32]. A higher cfPWV indicates greater arterial stiffness (i.e., a stiffer aorta) [33, 34] (all parameters are listed in Supplementary Material, Table 3). PWA measurement is followed immediately by cfPWV with two repeats of the measurement at an interval of 5–10 min. If the difference between the first and second cfPWV measurements is greater than 0.5 m/s, a third measurement will be performed [35].

#### Physical performance

Upper body strength is assessed using the arm curl and handgrip strength tests. Handgrip strength is assessed using a handheld dynamometer (Jamar Plus + , Sammons Preston Rolyon, USA) in a standing handshake position [36]. The test is conducted three times on both arms during which the participant squeezes the handle of the dynamometer with maximum effort, with one minute rest between each attempt [37]. The arm curl test is conducted using dumbbells (5 pounds (lbs) for females, 8 lbs for males) in a seated position with a straight back and feet firmly on the floor at should-width distance. Holding the dumbbell, the participant attempts to do as many bicep curls (elbow flexions) as possible within 30 s for each arm [38]. Upper body flexibility is assessed using the back scratch test [39]. In a standing position, participants reach as far as possible down to the middle of the back with one hand over the shoulder, fingers extended and palm facing the back. The other hand reaches up to the middle of the back as much as possible, palm facing out with fingers extended. The distance between the extended middle fingers of each hand is recorded. This test is then repeated with the other hand in the opposite position [38]. Physical performance is assessed using the 4-metre (4-m) walk, chair sit-to-stand, and 8-repetition maximal (8-RM) leg extension tests. In the 4-m walk test, the participant stands at the starting point, indicated on the floor, and when signalled, the participant walks at their normal pace until they reach the indicated end point. The walking area is indicated with a marker for the 4-m walk distance with a 1-m acceleration area [38]. Timing with a stopwatch begins when the command to go is given and stopped when the participant crosses the end point. The average of three attempts is taken. In the chair sit-to-stand test, the participant sits on a chair with the back straight and slight lean against the backrest, feet firmly on the floor at shoulder-width distance, and arms crossed with hands on opposite shoulders. Then, the participant stands up and sits down again. The total number of repetitions of standing up and sitting down on the chair within 30 s is recorded [38]. The 8-RM leg extension is assessed on a seated leg extension machine (640 LEC, GYMSPORTZ, Singapore) with an initial weight of 10 kg. If the participant can perform nine repetitions for a given weight (one set), the rate of perceived exertion (RPE) is asked after every set until 8-RM is achieved. If the participant can perform eight repetitions but not the ninth at a certain weight, that weight is recorded as the 8-RM strength. The 8-RM strength is recorded together with the RPE for the last set when 8-RM is achieved. A complete range of motion must be performed for a valid repetition.

#### Questionnaires

Participants complete several questionnaires about their lifestyle and health, including sociodemographics, measures of sleep (modified Pittsburgh Sleep Quality Index [40] and SATED questionnaires [41]), physical activity and sedentary behaviour by the Global Physical Activity Questionnaire (GPAQ) [42], cognitive performance by the Montréal Cognitive Assessment (MoCA) [43], frailty evaluation using the FRAIL scale [44], risk of malnutrition by the Mini Nutritional Assessment (MNA) [45], physical activity diary, and nutritional composition and dietary patterns using the three-day food record (two weekdays and one weekend day) [46] (Supplementary Material, Table 4).

### Digital biomarkers of ageing

#### Three-dimensional facial morphology

A three-dimensional (3D) image of the face, comprising up to 60,000 vertices per image, is captured using a 3D imaging system (Vectra®^H2^, Canfield Scientific, Inc., USA). Data capture is completed within five minutes per participant, who stands straight without any facial expressions on the markings of a positioning mat. Three images are taken on the left, front, and right sides of the face, which are then rendered into a 3D visualization by the Vectra®^H2^ software for subsequent morphological analyses, including wrinkles and anatomical landmarks [47]. Facial ages will be predicted by artificial intelligence models trained on chronological age and perceived age, respectively [47]. A grey-scale filter is applied over the 3D facial image to de-identify the participant.

#### Physical activity monitoring

The participants are issued an accelerometer (Fibion®, Fibion Inc., Jyväskylä, Finland) to monitor physical activity and sedentary behaviour for a minimum of three days up to seven days [48–50]. The participants wear the accelerometer positioned anteriorly in the front trouser pocket throughout the day [51], except during water-based activities and while sleeping at night. The device measures raw acceleration on three axes and can be used to estimate time and energy expenditure spent on different types of activities [51]. A physical activity diary is provided to the participants to record the time they go to bed, wake up, and any physical activity start and end times (Supplementary Material, Table 4).

### Statistical analyses

The proposed analyses will involve several statistical methods. Participants’ characteristics will be analysed according to age as both continuous variables and categorical variables (e.g., young, 21–44 years; middle-aged, 45–64 years; and older, 65 years and above). Subgroup analysis will be conducted based on sex, ethnicity (Chinese, Malay, or Indian), and physical activity status. Descriptive analyses will be performed with the *t*-test, analysis of variance, Mann–Whitney *U* test, Kruskal–Wallis test, Chi-square test, and Fisher’s exact test. Data that are not normally distributed will be considered for log-transformation if applicable. For continuous outcomes, linear regression models will be constructed to compute mean difference and 95% confidence intervals (CI). Binary logistic regression models will be constructed to compute odds ratios and 95% CI for results with binary outcomes. Data will be analysed by statistical analysis software (RStudio, Stata and SPSS). All statistical tests will be conducted at 5% level of significance. The associations between molecular (e.g., DNA methylation age, metabolic and inflammatory biomarkers in blood, saliva, and stool), physiological (e.g., body composition, physical performance, arterial stiffness, skin autofluorescence, cognitive status), and digital (e.g., facial age, objectively measured physical activity) biomarkers will be investigated both within and between groups (young, 21–44 years old; middle-aged, 45–64 years; and older, 65 years and above; male and female; Chinese, Malay, and Indian).

## Discussion

Findings from ABIOS have the potential to enhance understanding of the differences between chronological age and the phenotypes that change with biological age in Singaporeans. Singapore presents a unique demographic profile due to its multi-ethnic composition, which includes significant Chinese, Malay, and Indian sub-populations. This diversity provides a valuable opportunity to explore how different ethnic backgrounds might influence ageing processes and age-related health outcomes [52, 53].

Biological age is assessed in this study using DNA methylation age, a robust and standardized biomarker that has been used in clinical trials [54] and lifestyle intervention studies [55] to evaluate biological ageing and identify individuals who may benefit from targeted interventions [56]. In addition to biological age, physiological biomarkers, such as handgrip strength and routine laboratory markers, are well-established indicators of health status [57] and ageing, often used to monitor conditions like frailty and diabetes. These measures are readily available in clinical and primary care settings, making them practical for future implementation. Furthermore, lifestyle factors, such as physical activity and diet, play a crucial role in influencing biological age and healthspan [58–60] and are associated with a decreased risk of all-cause mortality and age-related chronic diseases [61, 62]. By collecting objective, quantitative data on physical activity and other lifestyle factors in this study, a clearer understanding of their effects on biological age and overall health status could be elucidated. This approach strengthens the translational potential of the study’s findings and supports the development of interventions for healthy ageing. Thus, the ABIOS study will build on this knowledge by exploring the molecular, physiological, and digital phenotypes associated with age and provide a deeper understanding of the characteristics in a Southeast Asian population.

Studies have shown that DNA methylation measures [63] and blood biochemistry biomarkers [64] of biological ageing indicate ethnic disparities in biological ageing, highlighting the need for more research on these measures in non-Western and non-Caucasian populations. Moreover, a study utilizing datasets from seven different ethnic groups found that epigenetic ageing rates are significantly associated with sex and ethnicity but did not adjust for ethnicity in their statistical models [52]. The existing gaps in medical knowledge pertaining to age-related chronic conditions in Asian populations and the differences in biomarkers of ageing phenotypes between Asian and Caucasian populations underscore the necessity of this research [65]. While direct comparisons with Caucasian populations are not part of the current study protocol, such analyses are valuable for identifying ethnic variations in biomarkers of ageing. Future work building on this study’s findings can include comparative analyses using publicly available datasets or collaborations with studies conducted in Caucasian or other ethnic populations.

By assessing various age-related biomarkers and physical functions as well as behavioural factors, the ABIOS study aims to describe the molecular, physiological, and digital phenotypes of individuals of Chinese, Malay, and Indian ethnicities in the Asian sub-populations. Establishing baseline data in this population is a necessary step to enable meaningful cross-ethnic comparisons and advance understanding of ageing biology globally.

## Supplementary Information

Below is the link to the electronic supplementary material.

Supplementary file1 (DOCX 74 KB)

## Data Availability

Since this is a protocol, no data generation or analyses have been performed in this article. For further inquiries, please contact the corresponding author.
