# Supplementary material for: Characterizing biomarkers of ageing in Singaporeans: the ABIOS observational study protocol
Source: GeroScience. 2025 Jan 17;47(3):3997–4008. doi: 10.1007/s11357-025-01511-1 (PMC12181143; doi:10.1007/s11357-025-01511-1)
Supplement: Supplementary file 1 — Supplementary file1 (DOCX 74 KB) [file 11357_2025_1511_MOESM1_ESM.docx]

**Supplementary Material**

Title:

**Characterizing biomarkers of ageing in Singaporeans: The ABIOS observational study protocol**

Jessica K Lu,^1,2^, Weilan Wang,^1,2^ Janjira Soh^1,2^, Elena Sandalova^1,2^, Zhi Meng Lim^1,2^, Santosh Kumar Seetharaman^3,4,5^, Jackie Jing-Dong Han^6,7^, Desmond Boon Seng Teo^3,8,9^, Brian K Kennedy^1,2,10,11^, Jorming Goh^1,2,11^, Andrea B Maier^1,2,3,12^

^1^ Centre for Healthy Longevity, National University Health System, Singapore, Singapore

^2^ Healthy Longevity Translational Research Program, Yong Loo Lin School of Medicine, National University of Singapore, Singapore, Singapore

^3^ Department of Medicine, Yong Loo Lin School of Medicine, National University of Singapore, Singapore, Singapore

^4^ Healthy Ageing Programme, Alexandra Hospital, Singapore, Singapore

^5^ Division of Geriatric Medicine, National University Hospital, Singapore, Singapore

^6^ CAS Key Laboratory of Computational Biology, CAS-MPG Partner Institute for Computational Biology, Shanghai Institute of Nutrition and Health, Chinese Academy of Sciences Center for Excellence in Molecular Cell Science, Collaborative Innovation Center for Genetics and Developmental Biology, Shanghai Institutes for Biological Sciences, Chinese Academy of Sciences, Shanghai, China

^7^ Peking-Tsinghua Center for Life Sciences, Academy for Advanced Interdisciplinary Studies, Center for Quantitative Biology (CQB), Peking University, Beijing, China

^8^ Division of Advanced Internal Medicine, National University Hospital, Singapore, Singapore

^9^ Chronic Programme, Alexandra Hospital, Singapore, Singapore

^10^ Department of Biochemistry, Yong Loo Lin School of Medicine, National University of Singapore, Singapore, Singapore

^11^ Department of Physiology, Yong Loo Lin School of Medicine, National University of Singapore, Singapore, Singapore

^12^ Department of Human Movement Sciences, @AgeAmsterdam, Faculty of Behavioural and Movement Sciences, Amsterdam Movement Sciences, Vrije Universiteit, Amsterdam, The Netherlands

**Correspondence to:** Prof Dr Dr Andrea B Maier, @Age, Department of Human Movement Sciences, Faculty of Behavioural and Movement Sciences, Vrije Universiteit Amsterdam, Amsterdam Movement Sciences, Van der Boechorstsraat 7, 1081 BT Amsterdam, the Netherlands

Email: a.b.maier@vu.nl

**Table 1. Assessments and parameters in biological samples.**

| Sample | Assessments | Parameters |
| --- | --- | --- |
| Blood | DNA methylation: EPIC chip v2.0 | DNAm age, years |
|  | Fasting haematological panel | Haemoglobin (Hb), g/dL |
|  |  | Total white cell count (WBC), x10^9^/L |
|  |  | Red cell count (RBC), x10^12^/L |
|  |  | Differential count |
|  |  | Neutrophils, x10^9^/L |
|  |  | Lymphocytes, x10^9^/L |
|  |  | Monocytes, x10^9^/L |
|  |  | Eosinophils, x10^9^/L |
|  |  | Basophils, x10^9^/L |
|  |  | Haematocrit (platelet count volume, PCV), au |
|  |  | Mean cell volume (MCV), fL |
|  |  | Mean corpuscular haemoglobin (MCH), pg |
|  |  | Mean corpuscular haemoglobin concentraiton (MCHC), g/dL |
|  |  | Red cell distribution width (RDW), % |
|  |  | Platelets, x10^9^/L |
|  | Fasting lipid profile | Total cholesterol, mmol/L |
|  |  | HDL cholesterol, mmol/L |
|  |  | LDL cholesterol, mmol/L |
|  |  | Cholesterol/HDL ratio, au |
|  |  | Triglycerides, mmol/l |
|  | Fasting glucose | Glucose, mmol/L  HbA1c, mmol/mol |
|  | Fasting liver profile | Total protein, g/L |
|  |  | Albumin, g/L |
|  |  | Globulin, g/L |
|  |  | A/G ratio, au |
|  |  | Total bilirubin, µmol/L |
|  |  | Alkaline phosphatase, U/L |
|  |  | Alanine aminotransferase (ALT), U/L |
|  |  | Aspartate aminotransferase (AST), U/L |
|  |  | Gamma-glutamyltransferase (GGT), U/L |
|  | Endocrine profile | Insulin, mU/L |
|  | Metabolite concentrations | Alanine, mmol/L |
|  |  | Glutamine, mmol/L |
|  |  | Glycine, mmol/L |
|  |  | Histidine, mmol/L |
|  |  | Isoleucine, mmol/L |
|  |  | Leucine, mmol/L |
|  |  | Valine, mmol/L |
|  |  | Phenylalanine, mmol/L |
|  |  | Tyrosine, mmol/L |
|  |  | Apolipoproteins A1/B, g/L |
|  |  | LDL cholesterol, mmol/L |
|  |  | HDL cholesterol, mmol/L |
|  |  | Remnant cholesterol, mmol/L |
|  |  | Total cholesterol, mmol/L |
|  |  | VLDL cholesterol, mmol/L |
|  |  | Total esterified cholesterol, mmol/L |
|  |  | Cholesterol esters (in HDL, LDL, VLDL), mmol/L |
|  |  | Docosahexaenoic acid, mmol/L |
|  |  | Linoleic acid, mmol/L |
|  |  | Monounsaturated fatty acids, mmol/L |
|  |  | Omega-3 fatty acids, mmol/L |
|  |  | Omega-6 fatty acids, mmol/L |
|  |  | Polyunsaturated fatty acids, mmol/L |
|  |  | Saturated fatty acids, mmol/L |
|  |  | Total fatty acids, mmol/L |
|  |  | Citrate, mmol/L |
|  |  | Glucose, mmol/L |
|  |  | Glycerol, mmol/L |
|  |  | Lactate, mmol/L |
|  |  | Pyruvate, mmol/L |
|  |  | Glycoprotein acetyls, mmol/L |
|  |  | 3-Hydroxybutyrate, mmol/L |
|  |  | Acetone, mmol/L |
|  |  | Acetoacetate, mmol/L |
|  |  | Acetate, mmol/L |
|  |  | Phosphatidylcholines, mmol/L |
|  |  | Phosphoglycerides, mmol/L |
|  |  | Sphingomyelins, mmol/L |
|  |  | Total choline, mmol/L |
| Saliva | Biobanking | future analysis |
|  | Metagenomic sequencing | Bacterial species and pathways |
| Stool | Biobanking | Future analysis |
|  | Metagenomic sequencing | Bacterial species and pathways |
|  | Short Chain Fatty Acids | Acetic acid (C2:0) |
|  |  | Propionic acid (C3:0) |
|  |  | Butyric acid (C4:0) |
|  |  | Isobutyric acid (C4:0i) |
|  |  | Valeric acid (C5:0) |
|  |  | Isovaleric acid (C5:0i) |
|  |  | Hexanoic acid (C6:0) |

 Adapted with relevant assessments from Sandalova, *et al*. [1]

**Table 2. Assessments for body composition, physical function, and activity tracking.**

| Test | Parameters, units |
| --- | --- |
| Dual energy X-ray absorptiometry (DEXA) for body composition | Left Arm Area, cm^2^  Left Arm Bone Mineral Content, g  Left Arm Bone Mineral Density, g/cm^2^  Right Arm Area, cm^2^  Right Arm Bone Mineral Content, g  Right Arm Bone Mineral Density, g/cm^2^  Left Rib Area, cm^2^  Left Rib Bone Mineral Content, g  Left Rib Bone Mineral Density, g/cm^2^  Right Rib Area, cm^2^  Right Rib Bone Mineral Content, g  Right Rib Bone Mineral Density, g/cm^2^  Thorax Spine Area, cm^2^  Thorax Spine Bone Mineral Content, g  Thorax Spine Bone Mineral Density, g/cm^2^  Lumbar Spine Area, cm^2^  Lumbar Spine Bone Mineral Content, g  Lumbar Spine Bone Mineral Density, g/cm^2^  Pelvis Area, cm^2^  Pelvis Bone Mineral Content, g  Pelvis Bone Mineral Density, g/cm^2^  Left Leg Area, cm^2^  Left Leg Bone Mineral Content, g  Left Leg Bone Mineral Density, g/cm^2^  Right Leg Area, cm^2^  Right Leg Bone Mineral Content, g  Right Leg Bone Mineral Density, g/cm^2^  Subtotal Area, cm^2^  Subtotal Bone Mineral Content, g  Subtotal Bone Mineral Density, g/cm^2^  Head Area, cm^2^  Head Bone Mineral Content, g  Head Bone Mineral Density, g/cm^2^  Total Area, cm^2^  Total Bone Mineral Content, g  Total Bone Mineral Density, g/cm^2^  Total Fat Mass, g  Total Lean + Bone Mineral Content (Fat-Free Mass), g  Total Mass, g  Total Fat Percentage, %  Abdominal Fat Mass, g  Abdominal Lean Mass + Bone Mineral Content, g  Abdominal Total Mass, g  Abdominal Fat Percentage, %  Hip/Buttock Fat Mass, g  Hip/Buttock Lean Mass + Bone Mineral Content, g  Hip/Buttock Total Mass, g  Hip/Buttock Fat Percentage, %  Total Body Fat Percentage, %  Estimated Visceral Adipose Tissue Mass, g  Estimated Visceral Adipose Tissue Volume, cm3  Estimated Visceral Adipose Tissue Area, cm^2^  Left Arm Bone Mineral Content, g  Left Arm Fat Mass, g  Left Arm Lean Mass, g  Left Arm Lean + Bone Mineral Content, g  Left Arm Total Mass, g  Left Arm Fat Percentage, %  Right Arm Bone Mineral Content, g  Right Arm Fat Mass, g  Right Arm Lean Mass, g  Right Arm Lean + Bone Mineral Content, g  Right Arm Total Mass, g  Right Arm Fat Percentage, %  Trunk Bone Mineral Content, g  Trunk Fat Mass, g  Trunk Lean Mass, g  Trunk Lean + Bone Mineral Content, g  Trunk Total Mass, g  Trunk Fat Percentage, %  Left Leg Bone Mineral Content, g  Left Leg Fat Mass, g  Left Leg Lean Mass, g  Left Leg Lean + Bone Mineral Content, g  Left Leg Total Mass, g  Left Leg Fat Percentage, %  Right Leg Bone Mineral Content, g  Right Leg Fat Mass, g  Right Leg Lean Mass, g  Right Leg Lean + Bone Mineral Content, g  Right Leg Total Mass, g  Right Leg Fat Percentage, %  Subtotal Bone Mineral Content, g  Subtotal Fat Mass, g  Subtotal Lean Mass, g  Subtotal Lean + Bone Mineral Content, g  Subtotal Total Mass, g  Subtotal Fat Percentage, %  Head Bone Mineral Content, g  Head Fat Mass, g  Head Lean Mass, g  Head Lean + Bone Mineral Content, g  Head Total Mass, g  Head Fat Percentage, %  Total Bone Mineral Content, g  Total Fat Mass, g  Total Lean Mass, g  Total Lean + Bone Mineral Content, g  Total Mass, g  Total Fat Percentage, %  Neck Area, cm^2^  Neck Bone Mineral Content, g  Neck Bone Mineral Density, g/cm^2^  Total Area, cm^2^  Total Bone Mineral Content, g  Total Bone Mineral Density, g/cm^2^  L1 Area, cm^2^  L1 Bone Mineral Content, g  L1 Bone Mineral Density, g/cm^2^  L2 Area, cm^2^  L2 Bone Mineral Content, g  L2 Bone Mineral Density, g/cm^2^  L3 Area, cm^2^  L3 Bone Mineral Content, g  L3 Bone Mineral Density, g/cm^2^  L4 Area, cm^2^  L4 Bone Mineral Content, g  L4 Bone Mineral Density, g/cm^2^  Total Area, cm^2^  Total Bone Mineral Content, g  Total Bone Mineral Density, g/cm^2^ |
| Handgrip strength | Left hand: strength, kg  Right hand: strength, kg |
| Gait speed – 4-metre walk | Time, seconds |
| Upper body strength – arm curl | Left arm: repetitions in 60 s  Right arm: repetitions in 60 s |
| Chair sit-to-stand test | Lower body strength: repetitions in 30 s |
| Back scratch test | Upper body flexibility: reach distance for Left and Right arms, cm |
| Leg extension strength | 8-Repetition Maximal, kg Rate of Perceived Exertion (RPE), scale from 6 (no exertion)–20 (maximal exertion) [2] |
| AGE reader Skin Autofluorescence Index | Skin autofluorescence, au (absorbance at 400 nm) |
| Facial ageing | 3D facial morphology |
| Physical activity motion tracking | 3-axis (forward, side-to-side, and vertical) accelerations, m/s ^2^ |

Adapted with relevant assessments from Sandalova, *et al*. [1]

**Table 3. Pulse wave analysis and pulse wave velocity parameters.**

|  | Assessments | Parameters, units |
| --- | --- | --- |
| Pulse wave analysis (PWA) | Brachial systolic and diastolic blood pressure | Resting blood pressure, mmHg |
|  | Central aortic systolic and diastolic blood pressure | Aortic systolic and diastolic blood pressure (SP and DP), mmHg |
|  | The difference between the maximum and minimum of the central pressure waveform (the height of the aortic pressure waveform or SP minus DP) | Aortic pulse pressure (PP), mmHg |
|  | The average aortic pressure in a pulse | Aortic mean arterial pressure (MAP), mmHg |
|  | Average heart rate in beats per minute during the measurement | Aortic heart rate (HR), beats per minutes (bpm) |
|  | The difference between two pressure peaks during systole. It is a measure of the wave reflected from lower body | Augmentation pressure (AP), mmHg |
|  | The ratio of AP to PP, in percentage | Augmentation Index (AIx), % |
|  | AIx normalized to a heart rate of 75 bpm | Augmentation Index 75 (AIx75), % |
| Pulse wave velocity (PWV): subtraction method | Carotid-femoral pulse wave velocity in metres/seconds, calculated as the distance in metre divided by pulse transit time in millisecond. PWV is averaged over the reading. | Pulse wave velocity (PWV), m/s |

Adapted with relevant parameters from Sandalova, *et al*. [1]

**Table 4: Questionnaires assessments and outcomes.**

| **Questionnaire** | **Assessment** | **Outcome** | **References** |
| --- | --- | --- | --- |
| Lifestyle & Health Survey | Socio-demographic information | Marital status  Living partners  Accommodation  Residency status  Country of birth and residence  Race  Education level  Smoking habits  Alcohol habits  Family history of lifespan  Work status  Occupation  Medical history/pre-existing conditions and concomitant medications  Diet and supplements | N.A. |
| Sleep (modified Pittsburgh Sleep Quality Index questionnaire + SATED questionnaire) | Sleep Quality  Sleep Health in 5 domains:  satisfaction  alertness  timing  efficiency  duration | Modified Pittsburgh Sleep Quality Index  Quantitative:   1. sleep duration [min score = 0 (better); max score = 3 (worse)] 2. sleep quality score [min score = 0 (better); max score = 3 (worse)]   SATED questionnaire  Quantitative: sleep health score  [min. score = 0 (poor sleep health); max. score = 10 (good sleep health)] | [3, 4] |
| Global Physical Activity Questionnaire (GPAQ) | Physical activity participation in three settings (domains) and sedentary behaviour: activity at work, travel to and from places, recreational activities | Quantitative: Total physical activity (metabolic equivalent (MET)-minutes/week)  Qualitative: inactive; minimally active; or health enhancing physical activity active | [5] |
| Montreal Cognitive Assessment (MOCA®) | Screening instrument for mild cognitive dysfunction by assessing different domains: attention and concentration, executive functions, memory, language, visuoconstructional skills, conceptual thinking, calculations, and orientation | Quantitative: MOCA total score (__/30)  Qualitative: Normal (≥26/30) | [6-9] |
| FRAIL questionnaire | Screening test to identify frail persons at risk of developing disability as well as decline in health functioning and mortality  Five components: fatigue, resistance, ambulation, illness, and loss of weight. | Quantitative: FRAIL total score (__/5)  Qualitative:  Robust (0 points)  Pre-fail (1–2 points)  Frail (3–5 points) | [10] |
| Mini-Nutritional Assessment (MNA) | A single, rapid assessment of nutritional status: to evaluate the risk of malnutrition to permit early nutritional intervention when needed based on food intake, weight loss, mobility, psychological stress or acute disease, neuropsychological problems, and body mass index (BMI) in the past three months | Quantitative: MNA total score (__/14)  Qualitative:  Normal nutritional status (12–14 points)  At risk of malnutrition (8–11 points)  Malnourished (0–7 points) | [11] |
| 7-day physical activity diary | The record of participants’ physical activity information for 7 days while wearing the Fibion and record each time when the Fibion is removed | Record of the participant’s physical activity patterns and for comparison with the 7-day objective physical activity measured using the Fibion activity tracking data | ABIOS protocol paper |
| 3-day food diary | Nutritional composition of diet, dietary patterns | Quantitative: macro-/ micro-nutrients  Qualitative: dietary patterns | N.A. |

Adapted with relevant assessments from Sandalova, *et al*. [1]

**References**

1. Sandalova E, Goh J, Lim ZX, Lim ZM, Barardo D, Dorajoo R, et al. Alpha-ketoglutarate supplementation and BiologicaL agE in middle-aged adults (ABLE)-intervention study protocol. Geroscience. 2023; <https://doi.org/10.1007/s11357-023-00813-6>

2. Borg G. Borg's Perceived Exertion And Pain Scales. 1998.

3. Buysse DJ, Reynolds III CF, Monk TH, Berman SR, Kupfer DJ. The Pittsburgh Sleep Quality Index: a new instrument for psychiatric practice and research. Psychiatry research. 1989.

4. Buysse DJ. Sleep health: can we define it? Does it matter? Sleep. 2014.

5. Committee IR. Guidelines for data processing and analysis of the International Physical Activity Questionnaire (IPAQ)-short and long forms. <http://www> ipaq ki se/scoring pdf. 2005.

6. Nasreddine ZS, Phillips NA, Bedirian V, Charbonneau S, Whitehead V, Collin I, et al. The Montreal Cognitive Assessment, MoCA: a brief screening tool for mild cognitive impairment. J Am Geriatr Soc. 2005; <https://doi.org/10.1111/j.1532-5415.2005.53221.x>

7. Nasreddine ZS, Phillips NA, Bédirian V, Charbonneau S, Whitehead V, Collin I, et al. The Montreal Cognitive Assessment, MoCA: a brief screening tool for mild cognitive impairment. Journal of the American Geriatrics Society. 2005.

8. Nasreddine ZS. Montreal Cognitive Assessment (MoCA) Administration and Scoring Instructions. 2010.

9. Julayanont P, Nasreddine ZS. Montreal Cognitive Assessment (MoCA): concept and clinical review. Cognitive screening instruments. Springer; 2017. p. 139-95.

10. Morley JE, Malmstrom TK, Miller DK. A simple frailty questionnaire (FRAIL) predicts outcomes in middle aged African Americans. J Nutr Health Aging. 2012; <https://doi.org/10.1007/s12603-012-0084-2>

11. Vellas B, Guigoz Y, Garry PJ, Nourhashemi F, Bennahum D, Lauque S, et al. The Mini Nutritional Assessment (MNA) and its use in grading the nutritional state of elderly patients. Nutrition. 1999; <https://doi.org/10.1016/s0899-9007(98)00171-3>
